# Place of work and residential exposure to ambient air pollution and birth outcomes in Scotland, using geographically fine pollution climate mapping estimates

**DOI:** 10.1016/j.envres.2015.05.010

**Published:** 2015-07

**Authors:** Chris Dibben, Tom Clemens

**Affiliations:** School of Geosciences, University of Edinburgh, Drummond Street, Edinburgh, UK

**Keywords:** Air pollution, Birth outcomes, Measurement error, Pollution climate mapping

## Abstract

**Objectives:**

A relationship between ambient air pollution and adverse birth outcomes has been found in a large number of studies that have mainly used a nearest monitor methodology. Recent research has suggested that the effect size may have been underestimated in these studies. This paper examines associations between birth outcomes and ambient levels of residential and workplace sulphur dioxide, particulates and Nitrogen Dioxide estimated using an alternative method – pollution climate mapping.

**Methods:**

Risk of low birthweight and mean birthweight (for *n*=21,843 term births) and risk of preterm birth (for *n*=23,086 births) were modelled against small area annual mean ambient air pollution concentrations at work and residence location adjusting for potential confounding factors for singleton live births (1994–2008) across Scotland.

**Results:**

Odds ratios of low birthweight of 1.02 (95% CI, 1.01–1.03) and 1.07 (95% CI, 1.01–1.12) with concentration increases of 1 µg/m^3^ for NO_2_ and PM_10_ respectively. Raised but insignificant risks of very preterm birth were found with PM_10_ (relative risk ratio=1.08; 95% CI, 1.00 to 1.17 per 1 µg/m^3^) and NO_2_ (relative risk ratio=1.01; 95% CI, 1.00 to 1.03 per 1 µg/m^3^). An inverse association between mean birthweight and mean annual NO_2_(−1.24 g; 95% CI, −2.02 to −0.46 per 1 µg/m^3^) and PM_10_ (−5.67 g; 95% CI, −9.47 to −1.87 per 1 µg/m^3^). SO_2_ showed no significant associations.

**Conclusions:**

This study highlights the association between air pollution exposure and reduced newborn size at birth. Together with other recent work it also suggests that exposure estimation based on the nearest monitor method may have led to an under-estimation of the effect size of pollutants on birth outcomes.

## Highlights

• Air pollution is associated with adverse pregnancy outcomes.
• NO_2_ , PM_10_ but not SO_2_ (now low in Scotland) showed significant associations.
• The magnitude of the effect appears to vary with different exposure methodologies.
• ‘Nearest monitor’ measurement method may have led to effect underestimation.

## 1 Introduction

It is now widely recognised, from studies in many different countries, that air pollution has adverse effects on human health (Beelen et al., 2014) and explains a significant proportion of the global burden of disease (Lim et al., 2012). Recent work has also suggested that air pollution may have a negative effect on the outcomes of pregnancy, such as low (<2500 g), very low (<1500 g) and mean birthweight and prematurity (Stieb et al., 2012). However, although these studies have enhanced knowledge and understanding of the risks of air pollution to foetuses, caution is still needed when interpreting the findings collectively because of important differences in methodological approaches (Woodruff et al., 2009). Importantly, the majority of studies use the nearest monitor method to estimate maternal exposure for both the entire pregnancy and trimester specific periods which averages pollutant concentrations from the nearest (static) monitor to the mother's residential location. These studies rely on daily or hourly changes in air pollution from a relatively small number of spatially diffused monitors that make up networks of pollution monitoring systems, to produce variation in the estimated individual level exposure (e.g. Ritz et al. (2000)). The mother's exposure will, therefore, be estimated from a monitor that is kilometres, or in some cases, tens of kilometres away from her residential location resulting in pollution exposure measurement error (Wilson et al., 2005).

Evidence from a recent multi-site study and two other studies have suggested that this methodology may have led to an underestimation in effect size. Stieb et al. (2012) and Dadvand et al. (2013) which base their multi-site/meta-analysis estimates on studies dominated by the nearest monitor methodology, have significantly lower effect sizes than Pedersen et al. (2013) a large multisite study, Hyder et al. (2014) and Lepeule et al. (2010) all of which utilise approaches that aim to capture spatial heterogeneity such as land use regression when estimating exposure. This raises important questions about the true effect sizes of air pollution on birth outcomes.

This paper sets out to assess whether earlier estimates of the effects of pollution on birth outcomes may have been underestimated in studies using a nearest monitor methodology by using a different approach. We used estimated annual pollution concentrations at a small area level estimated through a pollution climate mapping modelling approach. We also used workplace location together with hours spent at work to enhance our exposure estimate to include some variation in daily activity patterns.

## 2 Methods

### 2.1 Study population and birth outcomes data

The study population is drawn from the Scottish Longitudinal Study (SLS). The SLS is an approximately 5% sample of the Scottish national census as well as a number of other administrative data sources (Boyle et al., 2009). Linkages for this study were made between the SLS and maternity hospital admissions. This includes information on mothers age and usual place of residence (postcode) as well as smoking behaviour during pregnancy, parity, occupation of both the mother and father (where present) and information about the delivered baby including gestational age (estimated on the basis of both last menstrual period and ultrasound scan), birthweight, whether the baby was born in a singleton or multiple birth and its gender.

Data on singleton live births (years 1994 to 2008 inclusive and birth weights ranging from 500 to 6000 g) occurring to women in the SLS was extracted and the following birth outcomes analysed; risk of low birth weight (defined as infants born with a birth weight <2500 g) and mean birthweight for term births only and risk of moderately (33–37 weeks) and very (<33 weeks) preterm for all births. The distribution of these outcomes is presented in Table 1.

### 2.2 Exposure assessment

The study used modelled estimates of annual background concentrations of Sulphur Dioxide (SO_2_), Nitrogen Dioxide (NO_2_) and Particulate Matter less than 10 μm in diameter (PM_10_) in 1 km×1 km grid squares across Scotland. This data are published by the United Kingdom Atomic Energy Authority (AEA) (now Ricardo-AEA) and are used as the official indicators of air quality by the UK government (Brookes et al., 2011; NETCEN, 2005). These maps are derived using a pollution climate mapping (PCM) model approach. Briefly, this involves summing concentration values from a variety of sources including both large and small point sources as well as distant and area sources. For NO_2_, point source concentrations were estimated using emissions from the National Atmospheric Emissions Inventory (NAEI) and air dispersion modelling and distant sources were characterised from rural background monitor data. Area sources were modelled using a dispersion kernel and NAEI data (Brookes et al., 2011). A similar approach was used for PM_10_ though the heterogeneous composition of PM_10_ pollution required additional information, details of which can be found in Brookes et al. (2011, pp. 17–18).

We assumed that mothers lived at the postcode given at birth registration throughout her pregnancy. To estimate mothers' residential exposure to pollution, the location of the centroid of the postcode given at delivery was determined and the 1×1 km grid square from the PCM that it fell within was used to estimate exposure. However, estimating residential exposure alone may result in considerable exposure misclassification due to the lack of information about the daily activity patterns of pregnant women (Nethery et al., 2008). Thus, we examined two exposure models; residential exposure only and a combined residential and workplace (estimated in the same way based on postcode) exposure. In the latter, we used information recorded at the 2001 census on the number of hours on average spent at work per day to calculate a time-weighted exposure estimate combining both exposure locations. For the workplace exposure it was assumed that the place of work had not changed from the location recorded at the 2001 census. The final component of the exposure model was to correct for unmeasured national annual temporal variation in these modelled concentrations by including dummy terms for year of birth in the models.

### 2.3 Covariates

Given that the small area spatial contrast approach that we have adopted is considerably more susceptible to confounding (Strickland et al., 2009; Woodruff et al., 2009), it is necessary to have access to a wide range of contextual information. We therefore enhanced birth registration data through record linkage to census data. This is important because there is, for example, a well-established association between economic prosperity and birth outcomes (Moser et al., 2003) and people living in more polluted areas are likely to be poorer than those in less polluted areas (Mitchell and Dorling, 2003) leading to higher rates of adverse outcomes in polluted areas which may not be part of the pollution and birth outcomes pathway. We therefore measured potentially important confounders of the birth outcomes pollution relationship at the individual level. This included mother's age, parity, highest educational level, social class, ethnicity (distinguishing between south Asian and non-south Asian), lone parenthood, the season of birth and whether she smoked. We used an estimate of weekly wage based on occupational coding (Clemens and Dibben, 2014). If the mother's occupation was recorded, her gross weekly wage was added to that of the father if they were married or had registered the birth jointly. In order to equivalise the incomes of single and two parent births, an income equalisation multiplier of 1.6 was applied to the estimated wage of mothers of solely registered babies in a manner similar to that reported elsewhere (Dibben et al., 2006).

In light of important discussions that have been conducted elsewhere (Yap et al., 2012), we also carefully considered possible confounding by effects that vary at the area level and that may be correlated with both air pollution and birth outcomes. Given that we have adjusted for a large variety of confounding effects at the individual level, we would argue that the most aetiologically plausible source of any remaining genuinely area based effect arises from those characteristics of an area that could be considered stressful or may result in greater perceived sense of threat. Thus, rather than using a general, composite, measure of deprivation such as the Scottish Index of Multiple Deprivation (which is often used as a surrogate in the absence of adequate individual level information) which would likely result in over-adjustment (Schisterman et al., 2009) for individual and area based variation which is not confounding in this relationship, we restrict our area based effects to a measure of area crime rate (consisting of crimes of violence, sexual offences, domestic housebreaking, vandalism, drugs offences and common assault). This variable is measured at the mother's area of residence.

### 2.4 Statistical methods

Multilevel logistic, linear and multinomial regression models were fitted in STATA version 11 using the xtlogit, xtreg and mlogit with survey estimation programmes. The models were structured so that individuals were clustered within datazones (small areas of around 500–1000 people) to incorporate spatial dependence.

## 3 Results

Between 1994 and 2008, there were 21,843 singleton live term births and 23,086 total live births born to SLS sample members. The distribution of the birth outcomes and covariates is presented in Table 1. The mean estimated pollution exposures and the ranges and correlations between pollutants are shown in Table 2 for both residential exposures only and residential & workplace exposures combined. As would be expected, given the similarity between the main sources of emissions, mothers exposed to relatively high mean annual levels of NO_2_ were also likely to experience high levels of PM_10_. Pearson's correlation coefficient between the two was 0.81. SO_2_ was also correlated with both PM_10_ (0.58) and NO_2_ (0.39) and thus, similarly to other studies, these associations make it difficult to distinguish the effect of one pollutant from another in the models. Compared to NO_2_ and PM_10_, exposure to SO_2_ amongst the cohort was markedly lower on average.

Table 3 details the association between pollution and the risk of low birth weight less than 2500 g. SO_2_ was not a significant predictor of the risk of LBW in either the adjusted or unadjusted models though the point estimate was of a similar magnitude to NO_2_. Similarly to the models of mean birthweight, both PM_10_ and NO_2_ were highly significant in both adjusted and unadjusted models. Furthermore, there was little or no attenuation of the crude unadjusted effects with adjustment for other variables. The adjusted effects correspond to around a 233% and 211% increase in the risks of LBW across the range of values for PM_10_ and NO_2_ respectively. Again, inclusion of workplace exposure showed only negligible differences in the effect size when compared to residential exposure only.

Table 4 reports coefficients for the effect of both residential only exposure and the combined residential and workplace exposure to mean annual levels of NO_2_, PM_10_ and SO_2_ for mean birthweight. Exposure to all pollutants significantly reduced mean birthweight although the effect for SO_2_ was attenuated after adjustment for confounders. Adjusted PM_10_ and NO_2_ effects remained significant. Across the range of values for NO_2_ and PM_10_ these effects correspond to a reduction in mean birthweight of approximately 57 g and 94 g respectively after adjustment for confounders. There was a negligible reduction in the effect sizes when incorporating workplace exposure for all of the pollutants.

Table 5 reports findings from multinomial logit models predicting the risk of both moderately and very preterm birth and shows that in both adjusted and unadjusted models, none of the pollutants are associated with an increased risk of moderately preterm birth. Raised but not statistically significant associations were observed for the risk of very preterm birth in unadjusted models. Unlike the other outcomes, inclusion of workplace exposure resulted in a reduction (24%) in effect size for the risk of very preterm birth associated with PM_10_ exposure.

## 4 Discussion

The results from this study add to evidence supporting the negative effects of residential and workplace exposure to both NO_2_ and PM_10_ for foetal development, with a less significant effect observed for the risk of preterm birth. Effect sizes for foetal development outcomes are consistent across the different indicators (low birth weight and mean birthweight) and are robust to adjustment for potential confounders. They are also consistent when using a work and residence combined exposure estimate.

Table 6 summarises our findings and compares them to relevant effect estimates for the main meta-analysis in this area (Stieb et al., 2012), two major multisite studies (Dadvand et al., 2013; Pedersen et al., 2013), the most comparable personal monitoring based study (Jedrychowski et al., 2004) and a recent study based on satellite imaging for spatially detailed exposure estimation (Hyder et al., 2014). It shows that the effect sizes in this study appear to be relatively large when compared to the meta-analysis estimates from Stieb et al. (2012) and the multisite study from Dadvand et al. (2013) which are both based primarily on exposures estimated using a nearest monitor methodology. However, studies using more spatially refined personal monitoring or other spatial modelling techniques such as land use regression (LUR) or satellite imaging methods, have estimates much closer to this study (Hyder et al., 2014; Jedrychowski et al., 2004; Pedersen et al., 2013).

For the risk of preterm or spontaneous delivery, Table 6 shows that though not-significant at the 95% level (but significant at the 90% level) due to the low numbers of very preterm babies in our sample, the findings from our study for very preterm birth appear much higher than the estimates from the meta-analysis (Stieb et al., 2012). However, in contrast to our analysis, the majority of these studies used a measure of prematurity that differentiated between babies born before 37 weeks from those born at 37 weeks and after. Preterm birth can be difficult to estimate accurately as gestational age is often determined on the recall of last menstrual period. This is likely to result in a considerable amount of essentially random variation around the true gestational age, which will redistribute more true terms births into the preterm category, at this cut-off, than vice versa because of the higher number of term to preterm births in this period and therefore make it very difficult to detect a heightened risk of preterm (Been and Sheikh, 2013). One previous study, which used a similar measure of very and moderately preterm, appears to support this, reporting a significant 36% increase in the risk of very preterm birth with a 17 µg/m^3^ increase in the concentration of PM_10_. Furthermore, the risks for births between 32 and 36 weeks were insignificant for the same exposure (Suh et al., 2009). The low numbers and lack of statistical power means that our study can only hint at this interpretation but future research using larger and more powerful data sources should investigate this further.

A majority of previous studies have used the nearest monitor method for exposure estimation. If pollution concentration is spatially heterogeneous across the study area, the true pollution exposure of all subjects is approximately normal in distribution (ie fewer subjects are exposed to very low or very high concentrations) and subjects are not all located next to monitors or by chance the monitor located at a point where they measure *exactly* the mean exposure of all ‘their subjects’, then the nearest monitor studies will suffer from differential measurement error with a bias towards the null in any models using these estimates. Mothers exposed to a higher than mean level of pollution will on average have their exposure underestimated and vice versa. Given that many previous studies measure a mother’s exposure from a monitor up to 10 km away from her home, a space across which in an urban area there can be as much variation in pollution concentrations as exist across the whole study space, there may be a low correlation between the true and estimated pollution exposure and therefore a large bias in the estimated effect size towards the null (i.e. attenuation of the effect). In contrast, if techniques such as LUR or PCM, which capture greater spatial detail in pollution exposure estimates, are effectively capturing the temporal-average variance in pollution concentration across space, the main form of measurement error effecting the estimate, unmeasured temporal variance, will be independent of the pollution estimate (the error will be at random about the average exposure), and therefore Berkson in type. Even if large, though lowering the precision of any model estimates, Berkson error will not bias the effect estimates. The explanation for the higher effects sizes observed in this study may lie, at least in part, with the type of measurement error effecting the nearest monitor methodology and the similarly large effect sizes in the few more recent studies using methods other than the nearest monitoring station (Jedrychowski et al., 2004; Pedersen et al., 2013). Future studies, should look to test this possibility explicitly by comparing effect estimates from both approaches for the same dataset.

This paper explored miss-measurement due to daily activity patterns. The similarity in findings, when estimating pollution exposure through [1] residential and [2] residential and work location combined, raises a couple of points for discussion. Firstly, it suggests that an estimate of exposure through residence may be a good proxy for both residential and workplace exposure. Secondly, the lack of attenuation in effect size, once workplace is included, provides further evidence that the pollution-birth outcome relationship is not due to unmeasured confounders. Exposure to pollution at work is far more likely to be at random. If the effect estimates based on residence only were confounded we would expect a greater degree of attenuation when compared to effect sizes based on workplace and residence combined.

Exactly how air pollutants interfere with foetal development and preterm birth is not well understood (Lacasana et al., 2005). Several biological mechanisms have been proposed for foetal development, including disturbances of the uterine blood flow, disturbances of the pituitary–adrenocortico-placental system, and increased maternal susceptibility to infections (Ritz and Yu, 1999). Several factors have been identified that may cause disturbances to the uterine blood flow. These include inflammation of the airways associated with air pollution that may alter the umbilical and placental blood flow, reducing the exchange of nutrients and thereby affecting foetal development (Lacasana et al., 2005). Additionally, studies have suggested that DNA adduct levels in maternal blood and placentas are higher in areas of pollution leading to potential decreases in the exchange of oxygen and nutrients (Šram et al., 2005). Disturbances to the pituitary–adrenocortico-placental system result from the anti-oestrogenic effects of exposure, which can disrupt the endocrine system (Šram et al., 2005). Although air pollution does not directly cause maternal infections, respiratory infections associated with the inhalation of air pollutants have been suggested as a causal factor for preterm birth (Lacasana et al., 2005). In particular, exposure to specific pollutants may impair immune function and thereby enhance susceptibility to infection (Sagiv et al., 2005). Genetic factors have also been implicated with the presence of glutathione S-transferases mu 1 (GSTM1) null genotypes, which reduce metabolic detoxification, being associated with an increased risk of preterm birth through an increased susceptibility to air pollution (Suh et al., 2008).

This study has limitations. Pollution exposure was estimated using PCM modelled pollution concentrations and these will differ from actual personal exposures. However, compared with actual mechanical observations from a variety of spatially dispersed monitoring stations, the fit of the data, for NO_2_ in particular, is very good (Walker et al., 2011) and as argued above, measurement error associated with a spatially modelled method such as PCM is likely to be Berkson, which though leading to an increase in residual variance will not result in attenuation of the effect size. No account could be taken of variations over time or occasional spike pollution events that might have an extra health impact. It is therefore not possible to say that the negative health impact of an area with a high mean rate may result from chronic constant exposure or high ‘pulses’ of pollution or some combination of the two.

The individual effects of each of the pollutants should not be over interpreted in this study given the high degree of spatial correlation between pollutants. PM_10_ and NO_2_, in particular, are highly correlated and it is therefore possible that some of the effect for PM_10_ is related to NO_2_ and vice versa. The high level of correlation also meant that a multi-pollutant model could not partition the effect by pollutant type. In contrast to findings in this paper other studies have found significant effects for SO_2_ (Bobak, 2000; Lin et al., 2004). This difference is probably because of the generally low levels of SO_2_ in Scotland during the study period. The non-significant finding in this study should therefore not be interpreted as evidence of no effect. We did not adjust for maternal height as, although it was available, it contained a substantial number of missing cases (~4000) and, when conducting sensitivity analysis on the complete case subset with adjustment for height, no differences in the pollution effects were found. There was no available information about mode of delivery (meaning that we were unable to restrict the preterm analysis to spontaneous births), maternal exposure to passive smoke or maternal pre-pregnancy weight so we were unable to adjust the models for these potentially confounding effects in the models. Location information was derived from postcode rather than precise address point. Individuals living in postcodes straddling a grid square boundary may, therefore, have been assigned an incorrect pollution value. However because the measure of pollution is area based and therefore a spatially smoothed measure, the scale of miss measurement is likely to be small. As residential location was only recorded at birth, we were also not able to identify mothers who moved during pregnancy. This may have introduced classical measurement error, potentially biasing the effect towards the null.

In conclusion, this study adds to growing evidence for a link between maternal exposure to outdoor ambient air pollution and negative pregnancy outcomes. The results suggest that that effect sizes in many previous studies may have been underestimated. The findings support the importance of using spatially disaggregated pollution exposure data and highlights possible problems associated with the estimation of gestational age when determining prematurity. The findings are strengthened by a number of design and analytical features including the use of small areas for pollution exposure, the incorporation of workplace time and location into pollution exposure estimation and adjustment for a large number of potentially confounding effects.

## Funding

Tom Clemens was supported by a grant from the Wellcome Trust (The Scottish Health Informatics Programme-Ref WT086113) and the Farr Institute – Scotland.

## Competing financial interests

The authors declare they have no actual or potential competing financial interests.

## Figures and Tables

**Table 1:** Descriptive statistics for the SLS sample for both outcomes and covariates.

**Categorical variables**	**Term births**		**Preterm births**	
	***N***	**%**	***N***	**%**
**Low birthweight (<2500 g)**				
Yes	457	2.09	n/a	
No	21,428	97.91	n/a	
			
**Prematurity**				
Less than 32 weeks	n/a		193	0.83
Between 32 and 36 weeks inclusive	n/a		1,049	4.54
Greater than 36 weeks	n/a		21,885	94.63
				
**Social class**				
Professional	935	4.27	972	4.2
Managerial and Technical	5816	26.58	6092	26.34
Skilled Non-manual	7534	34.43	7,971	34.47
Skilled Manual	1857	8.49	1961	8.48
Partly Skilled	3,500	15.99	3741	16.18
Unskilled	934	4.27	993	4.29
Armed Forces	<15	<0.1	<15	<0.1
Unemployed	1302	5.95	1,390	6.01
				
**Parity**				
Multiparous	12,570	57.44	13,176	56.97
Nulliparous	9315	42.56	9951	43.03
				
**Smoker during pregnancy**				
No	16,712	76.36	17,548	75.88
Yes	5173	23.64	5579	24.12
**Ethnicity**				
Non-South Asian	21,614	98.76	22,843	98.77
South Asian	271	1.24	284	1.23
				
**Mothers age at delivery**				
17–18	546	2.49	579	2.5
19–23	3070	14.03	3257	14.08
24–28	5987	27.36	6322	27.34
29–33	7303	33.37	7711	33.34
34–38	4078	18.63	4291	18.55
39+	901	4.12	967	4.18
				
**Education**				
None	3008	13.74	13.95	13.95
O’ grade, Standard grade or equivalent	4922	22.49	5237	22.64
Higher, ‘A’ level, AS level or equivalent	1671	7.64	1768	7.64
GSVQ/SVQ Level 1 or 2 or equivalent	4179	19.1	4420	19.11
GSVQ/SVQ Level 3, ONC, OND or equivalent	1068	4.88	1128	4.88
HNC, HND, SVQ Level 4 or 5 or equivalent	2059	9.41	2150	9.3
First degree or higher degree or equivalent	1714	7.83	1786	7.72
Professional Qualifications or equivalent	3264	14.91	3412	14.75
				
**Season of birth**				
Winter	5094	23.28	5412	23.4
Spring	5539	25.31	5841	25.26
Summer	5624	25.7	5967	25.8
Autumn	5628	25.72	5907	25.54
				
**Lone mother**				
No	20,309	92.8	21,423	92.63
Yes	1576	7.2	1704	7.37
				
**Continuous variables**	**Mean**			

**Birthweight (g)**	3481.22		n/a	
**Crime Rate (Log scaled)**	6.00		6.00	
**Estimated weekly wage (£)**	353.34		352.27	
**Gestational age (weeks)**	39.68		39.37	

**Table 2:** Summary measures of air pollution concentrations for recorded births (1994–2008) in 1×1 km grid square for both residence only and residence and workplace exposure combined.

	***N***	**Mean**	**SD**	**Min**	**Max**	**Range**	**Pearson's correlation coefficients**	
							**PM**_**10**_**(µg/m^3^)**	**SO**_**2**_**(µg/m^3^)**
**Term births only (Residential)**								
PM_10_ (µg/m³)	21843	13.30	2.54	6.12	23.90	17.77	1.00	
SO_2_ (µg/m³)	21843	5.41	5.12	0.00	37.26	37.26	0.58	1.00
NO_2_ (µg/m³)	21843	17.47	10.0	0.73	57.95	57.22	0.81	0.39
								
**All births (Residential)**								
PM_10_ (µg/m³)	23086	13.30	2.54	6.12	23.90	17.77	1.00	
SO_2_ (µg/m³)	23086	5.41	5.11	0.00	37.26	37.26	0.59	1.00
NO_2_ (µg/m³)	23086	17.48	9.89	0.73	57.95	57.22	0.81	0.40
								
**Term births only (Residential and workplace)**								
PM_10_ (µg/m³)	21839	13.37	2.53	6.17	23.17	17	1.00	
SO_2_ (µg/m³)	21839	5.44	5.05	0	35.73	35.73	0.58	1.00
NO_2_ (µg/m³)	21839	17.80	9.87	0.73	56.81	56.08	0.81	0.39
								
**All births (Residential and workplace)**								
PM_10_ (µg/m³)	23079	13.37	2.52	6.17	23.90	17.73	1.00	
SO_2_ (µg/m³)	23079	5.44	5.04	0	37.26	37.26	0.59	1.00
NO_2_ (µg/m³)	23079	17.80	9.85	0.73	57.95	57.22	0.81	0.39

**Table 3:** Crude and adjusted models [OR (95% CI)] predicting the risk of low birth weight (<2500 g) for term births^a^.

	**Crude**^b^	**Adjusted for other predictors**^c^
**Pollutant (Residential)**		
PM_10_ (µg/m³)	1.12⁎⁎⁎ (1.07,1.19)	1.07⁎⁎ (1.01,1.14)
NO_2_ (µg/m³)	1.03⁎⁎⁎ (1.02,1.04)	1.02⁎⁎⁎ (1.00,1.03)
SO_2_ (µg/m³)	1.03⁎⁎ (1.01,1.06)	1.02⁎ (1.00,1.05)
		
**Pollutant (Residential and workplace)**		
PM_10_ (µg/m³)	1.12⁎⁎⁎ (1.06,1.18)	1.07⁎⁎ (1.01,1.13)
NO_2_ (µg/m³)	1.02⁎⁎⁎ (1.01,1.04)	1.01⁎⁎ (1.00,1.03)
SO_2_ (µg/m³)	1.03⁎⁎ (1.01,1.06)	1.02 (0.99,1.05)

⁎ (*p*<.10).

⁎⁎ (*p*<.05).

⁎⁎⁎ (*p*<.01).

a Term births were defined as those occurring after 36 completed weeks.

b Adjusted for year of birth and gestational age.

c Adjusted for social class, parity, individual estimated income, ethnicity, smoking, area log crime rate, mother's age, mothers education, season of birth, lone parent at birth registration and year of birth.

**Table 4:** Crude and adjusted models [coefficients (95% CI)] predicting mean birth weight for term births^a^.

	**Crude**^b^	**Adjusted for other predictors**^c^
**Pollutant (Residential)**		
PM_10_ (µg/m³)	−12.15⁎⁎⁎ (−15.81,−8.48)	−5.27⁎⁎⁎ (−8.84,−1.69)
NO_2_ (µg/m³)	−2.37⁎⁎⁎ (−3.12,−1.61)	−0.99⁎⁎⁎ (−1.72,−0.25)
SO_2_ (µg/m³)	−3.11⁎⁎⁎ (−4.93,−1.29)	−1.34 (−3.07,0.40)
		
**Pollutant (Residential and workplace)**		
PM_10_ (µg/m³)	−10.82⁎⁎⁎ (−14.51,−7.12)	−4.82⁎⁎⁎ (−8.42,−1.21)
NO_2_ (µg/m³)	−2.13⁎⁎⁎ (−2.89,−1.37)	−0.92⁎⁎ (−1.66,−0.18)
SO_2_ (µg/m³)	−3.11⁎⁎⁎ (−5.00,−1.22)	−1.27 (−3.07,0.54)

^⁎^(*p*<.10).

⁎⁎ (*p*<.05).

⁎⁎⁎ (*p*<.01).

a Term births were defined as those occurring after 36 completed weeks.

b Adjusted for year of birth and gestational age.

c Adjusted for social class, parity, individual estimated income, ethnicity, smoking, area log crime rate, mother’s age, mothers education, season of birth, lone parent at birth registration and year of birth.

**Table 5:** Crude and adjusted multinomial logistic models [Relative risk ratio (95% CI)] predicting risk of very (<32 wks) and moderately (32–36 wks) preterm birth. Both compared to the base category of term births (>36 wks).

	**Crude**^a^	**Adjusted for other predictors**^b^
**Pollutant (Residential)**		
PM_10_ (µg/m³)		
Mod Preterm	1.01 (0.97,1.04)	0.99 (0.96,1.03)
Very Preterm	1.10^⁎⁎^ (1.02,1.18)	1.08^⁎^ (1.00,1.17)
NO_2_ (µg/m³)		
Mod Preterm	1.00 (0.99,1.01)	1.00 (0.99,1.00)
Very Preterm	1.02^⁎⁎^ (1.01,1.03)	1.013 (1.00,1.03)
SO_2_ (µg/m³)		
Mod Preterm	1.00 (0.99,1.02)	1.00 (0.98,1.02)
Very Preterm	1.03 (0.99,1.07)	1.02 (0.98,1.07)
		
**Pollutant (Residential and workplace)**		
PM_10_ (µg/m³)		
Mod Preterm	1.00 (0.97,1.04)	0.99 (0.96,1.03)
Very Preterm	1.09^⁎⁎^ (1.02,1.18)	1.08^⁎^ (0.99,1.16)
NO_2_ (µg/m³)		
Mod Preterm	1.00 (0.99,1.01)	1.00 (0.99,1.00)
Very Preterm	1.02^⁎^ (1.00,1.03)	1.01 (0.99,1.03)
SO_2_ (µg/m³)		
Mod Preterm	1.00 (0.99,1.02)	1.00 (0.98,1.02)
Very Preterm	1.02 (0.99,1.07)	1.02 (0.98,1.06)

*Source*: Scottish Longitudinal Study." as the table footnote. ^⁎^(*p*<.10), ^⁎⁎^ (*p*<.05), ^⁎⁎⁎^(*p*<.05).

a Adjusted for year of birth and gestational age.

b Adjusted for social class, parity, individual estimated income, ethnicity, smoking, area log crime rate, mother's age, mothers education, season of birth, lone parent at birth registration and year of birth.

**Table 6:** Summary table of effect estimates from the main recent meta-analysis, multisite and comparable personal monitoring study compared to the estimates in the present study.

**Study**	**Type**	**Pollutants**	**Exposure method**	**Effect (scaled to per 10 µg/m^3^ to aid comparison)**		
				**Birthweight (linear coefficients, (g))**	**Risk of LBW (Odds ratio)**	**Risk of Prematurity (Odds ratio and relative risk ratio)**
Stieb et al. (2012)	Meta-analysis	PM_10_, NO_2_	Nearest monitor	PM_10_: −8.50	PM_10_: 1.05	PM_10_: 1.16
				NO_2_: −7.50	NO_2_: 1.01	NO_2_: 1.04
Dadvand et al. (2013)	Multisite single study	PM_10,_ PM_2.5_	Predominantly Nearest monitor	PM_10_: −8.90	PM_10:_ 1.03	Not available
					PM_2.5:_ 1.10	
Pedersen et al. (2013)	Multisite single study	PM_10_, NO_2_, PM_2.5_	LUR	Not available	PM_10_: 1.16	Not available
					NO_2_: 1.09	
					PM_2.5_: 1.39	
					PM_2.5_ (for women exposed to <20 µg/m^a^): 1.99	
Jedrychowski et al. (2004)	Single Study	PM_2.5_	Personal monitoring	PM_2.5_: −35.00	Not available	Not available
Hyder et al. (2014)	Single Study	PM_2.5_	Satellite imaging	PM_2.5_: −79.25	PM_2.5_: 1.43	PM_2.5_: 1.00
Present study	Single Study	PM_10_, NO_2_	PCM	PM_10_: −52.7	PM_10_: 1.97	PM_10_ mod preterm: 0.90
				NO_2_: −12.4	NO_2_: 1.22	PM_10_ very preterm: 2.16
						NO_2_ mod preterm: 1.00
						NO_2_ very preterm: 1.10

a This range is the most similar to the actual distribution of PM_2.5_ in Scotland.
